# Three-Dimensional Macroporous rGO-Aerogel-Based Composite Phase-Change Materials with High Thermal Storage Capacity and Enhanced Thermal Conductivity

**DOI:** 10.3390/ma16134878

**Published:** 2023-07-07

**Authors:** Zhang Tao, Wei He, Xiaoliang Xu, Jianzhong Fan, Zhifeng Zhang, Ziyue Yang, Yanqiang Liu, Heng Ma, Miao Qian, Mu Yang

**Affiliations:** 1 GRINM Metal Composites Technology Co., Ltd., Beijing 101407, China; 2 Wuhan NARI Limited Liability Company, State Grid Electric Power Research Institute, Wuhan 430074, China; 3 Beijing Advanced Innovation Center for Materials Genome Engineering, Beijing Key Laboratory of Function Materials for Molecule & Structure Construction, School of Materials Science and Engineering, University of Science and Technology Beijing, Beijing 100083, China; 4 Zhejiang Huadian Equipment Testing Institute Co., Ltd., Hangzhou 310015, China

**Keywords:** macroporous rGO aerogel, phase-change materials, thermal energy storage, thermal conductivity, pore structure

## Abstract

Three-dimensional porous network encapsulation strategy is an effective means to obtain composite phase-change materials (PCMs) with high heat storage capacity and enhanced thermal conductivity. Herein, macroporous reduced graphene oxide (rGO) aerogels with adjustable pore size are prepared by the emulsion template method and hydrothermal reduction process. Further, the shape-stabilized rGO-aerogel-based composite PCMs are constructed after the combination of 3D porous rGO supports and paraffin wax (PW) through vacuum melting infiltration. By regulating the pore structure of the rGO aerogel network, the rGO-based composite PCMs achieve excellent energy storage properties with a phase-change enthalpy of 179.94 J/g for the loading amount of 95.61 wt% and an obvious enhancement in thermal conductivity of 0.412 W/m^−1^·K^−1^, which is 54.89% higher than pristine PW and enduring thermal cycling stability. The obtained macroporous rGO-aerogel-based composite PCMs with high thermal storage and heat transfer performance effectively broaden the application of PCMs in the field of thermal energy storage.

## 1. Introduction

The depletion of fossil fuels and their inefficient utilization are impeding the development of society; developing advanced energy storage techniques is regarded as an effective way to resolve energy shortages and increase the efficiency of energy use. Thermal energy storage (TES) is one of the crucial technologies to support the development of renewable energy [1]. As a potential pollution-free, convenient, and efficient thermal energy storage material, phase-change materials (PCMs) play an important role in overcoming the intermittent defects and mismatches of energy supply and demand through the absorption and release of latent heat [2,3]. The extensive research on PCMs and their applications effectively alleviates the contradiction between resources and development. Typically, the PCMs are mainly used in the field of thermal storage and thermal management, such as waste heat recovery [4,5], energy-saving buildings [6], infrared stealth [7], peak regulation in power systems, thermal dissipation of electronic devices and battery thermal control system [8,9,10], etc.

Organic PCMs achieving thermal energy storage by solid–liquid phase transition processes are widely investigated and applied due to the advantages of high thermal storage capacity, less phase separation, limited volume change, chemical stability, and non-toxicity. However, the deficiency of leakage during the solid–liquid phase change period and low intrinsic thermal conductivity severely restrict the application of organic PCMs. Therefore, obtaining shape-stabilized composite PCMs with excellent thermal transfer performance is becoming the focus of research. To avoid the leakage of PCMs as the phase transition happens, porous supporting matrices such as diatomite [11], metal foam [12], polymer networks [13], porous carbon materials, and porous biomasses are commonly implemented to encapsulate the PCMs [14,15,16]. A sufficient capillary force and van der Waals force between the supporting matrix and PCMs effectively restrict the flow of liquid PCMs. Among the supporting matrices used, carbon-based porous materials have received extensive attention and investigation due to their relatively low density and excellent compatibility with organic PCMs. For instance, Chen et al. obtained the CNT/eicosane composite PCMs via colloid aggregation by adding calcium chloride and achieving a high thermal storage density of 204.8 J/g [17]. Maleki et al. constructed a three-dimensional interconnected porous polystyrene–CNT foam scaffold, and the obtained shape-stabilized paraffin/polystyrene–CNT composites exhibited high PCM loading of 82.5% and latent heat of 119.3 J/g [18]. Fang et al. built a crosslinking graphene skeleton via a hydrothermal process and prepared graphene/polyethylene glycol composite PCMs with a phase-change enthalpy of 223.2 J/g [19]. However, not only the shape-stability performance but also the enhanced thermal conductivity is a key indicator for the wider application of composite PCMs. To date, adding high thermal conductivity fillers to PCMs is the most direct method to improve the thermal conductivity, yet in fact, the enhancement in thermal conductivity by a small amount of filler is limited and excessive addition would greatly reduce the heat storage capacity of the composite PCMs. Thus, achieving high heat storage density and fast thermal conduction at the same time is the bottleneck of the development of composite PCMs. For the three-dimensional network-based composite PCMs, relatively low density, high porosity, and good compatibility of porous supports are the basis for obtaining high heat storage capacity. In addition, the formation of a continuous and rapid thermal conduction pathway is the key to the improvement in the thermal conductivity of composite PCMs. Constructing a 3D continuous porous network supporting matrix with low density, fast thermal transfer, and good compatibility with PCMs is regarded as a valid strategy for the simultaneous high thermal storage and conduction property of the composite PCMs. In carbon-based materials, graphene is widely used to construct 3D continuous networks or aerogel-supporting matrices due to its high in-plane thermal conductivity, low density, good compatibility with organic PCMs, and two-dimensional structural characteristics, which can load the PCMs in the pores of aerogels steadily and form a rapid continuous heat transfer path in the composite PCMs [20,21]. The pore structure and pore size distribution of graphene aerogel (GA) supports often have a great influence on the heat storage and thermal conductivity of the composite PCMs. For the macroporous GA supports, although very large pore size and porosity can load sufficient PCM, the increase in thermal conductivity will be limited due to the discontinuous pore structure and less heat transfer path, and meanwhile, the PCM leakage will occur during the phase transition period due to insufficient capillary force between PCM and supports. Conversely, excessive small pore size and porosity can rapidly improve the heat transfer performance of the composite PCMs, but the heat storage density will decrease due to the reduction in the load capacity of the PCM. Therefore, achieving a high proportion of PCM loading alongside maximum thermal conductivity improvement by regulating the pore structure and pore size of GA in the macroporous range is worth studying but rarely reported.

Here, we skillfully use Pickering emulsion stabilized by the graphene oxide (GO) nanosheet as a soft template to construct a three-dimensional GA network, and meanwhile, the GO content in the emulsion is changed to regulate the pore structure of the aerogel support. Subsequently, shape-stabilized composite PCMs are prepared after combining the PCMs and GA supports via the vacuum melting infiltration method. Based on the construction of GA supports with adjustable macroporous structure and continuous three-dimensional thermal conductivity path, GA-based composite PCMs with high thermal storage capacity and greatly enhanced thermal conductivity are obtained, which provides a reference for the construction of macroporous network-based composite PCMs and expand their potential application in the thermal energy storage field.

## 2. Experimental Section

### 2.1. Materials

The graphite was purchased from Saen Chemical Technology Co. (Shanghai, China). Chemical reagents, including potassium permanganate (KMnO_4_), cyclohexane (CYH), sodium nitrate (NaNO_3_), sulfuric acid (H_2_SO_4_, ~98 wt%), hydrogen peroxide (H_2_O_2_, 30 wt%), and ethanol, were obtained from Aldrich Company (Saint Louis, MO, USA) and used without further purification. The ascorbic acid (AsA) and paraffin wax (PW, density of ~0.9 g/cm^3^ at 20 °C) were provided by Sinopharm Chemical Reagent Co. (Shanghai, China). Ultra-pure deionized water (18.2 MΩ·cm^−1^) was supplied by a Master system (Sega, Shinagawa, Tokyo).

### 2.2. Preparation of Three-Dimensional Macroporous rGO Aerogel

The graphene oxide nanoplates (GO) were prepared by Hummer’s method [22]. In a typical procedure, 60 mL of concentrated sulfuric acid was added to the three-necked flask and kept in an ice bath at 0 °C for 30 min. Then, 1 g of NaNO_3_ and 2 g of flake graphite were added to the flask and stirred for 15 min. Next, 8 g of KMnO_4_ was slowly added to the container and continuously reacted for 30 min, and the reaction was carried out for 2 h at 40 °C to obtain a viscous brown-yellow solution. After that, the temperature of the system was raised to about 98 °C; simultaneously, 45 mL of deionized water was dropped into the three-necked flask. When the reaction was completed, 30 mL of hydrogen peroxide was added to the solution, and then we washed the centrifugal sediment several times until the system became neutral. Finally, the hetero-ion and water molecules in the solution were removed by dialysis. After freeze-drying the product, GO nanoplates were obtained.

The obtained GO nanosheets with masses of 60 mg, 70 mg, 80 mg, and 90 mg were added into 10 mL water solvents, respectively, for ultrasonic dispersion. Different amounts of ascorbic acid (300 mg, 350 mg, 400 mg, 450 mg) were added to the above solutions as reducing agents, the ratio of ascorbic acid to GO remained 5:1. Then 10 mL cyclohexane was added as emulsifier, and the stable emulsion structure was formed by stirring with an emulsifying agitator at 10,000 rpm for 1 h. The rGO hydrogel was prepared by hydrothermal reaction of the above emulsion at 70 °C for 6 h. Finally, the rGO hydrogel was washed with deionized water and ethanol, and then freeze-drying was conducted to obtain the three-dimensional macroporous rGO aerogel. The obtained aerogels were named rGA-6, rGA-7, rGA-8, and rGA-9 according to the amount of GO added.

### 2.3. Preparation of rGO/PW Composite PCMs

The paraffin wax (PW) was encapsulated into rGO aerogel by vacuum infiltration strategy. Firstly, the PW was melted in a vacuum at 80 °C. Then the aerogel of rGA-6, rGA-7, rGA-8, and rGA-9 were immersed in melting PW and kept in a vacuum at 80 °C for 8 h to achieve saturated adsorption, respectively. After that, the excess PW adhered on the three-dimensional aerogel surface was removed by filter paper until the composites stabilized and, thus, a series of composite phase-change materials (CPCMs) of rGA-6/PW, rGA-7/PW, rGA-8/PW, and rGA-9/PW were obtained. The mass fraction of PW in CPCMs can reach 88%, 93%, 95%, and 96%, corresponding to rGA-6, rGA-7, rGA-8, and rGA-9 three-dimensional rGO aerogels, respectively. The calculation formula is shown in Equation (1) [23]:

(1) $$\omega =1-\frac{m_{a}}{m_{c}}$$

where ω is the mass fraction of PW in CPCMs, $m_{a}$ represents the mass of rGO aerogel, and $m_{c}$ is the mass of the CPCMs.

### 2.4. Characterizations

The morphology and microstructures of the samples were characterized by field-emission scanning electron microscopy (SEM, Hitachi SU8000, Tokyo, Japan) and a transmission electron microscope (TEM, JEM-100CX, Tokyo, Japan). The chemical structures of the samples were measured using Fourier-transform infrared spectroscopy (FI-IR, Nicolet 6700 by the KBr pellet technique). The phase structure of the samples was characterized by an X-ray diffractometer (M21X, Cu Kα radiation, λ = 1.541 Å) over an angular range of 10–80° (2θ). The thermal stability was evaluated using a synchronous thermal analyzer (TGA, Netzsch STA449F3, Selb, Germany) at a heating rate of 10 °C/min under a flowing nitrogen atmosphere. The pore diameter distributions and accumulated pore area distribution of samples were measured by an automatic mercury injection apparatus (Mack corporation Auto Pore Ⅳ 9510, Atlanta, GA, USA). Differential scanning calorimetry (DSC, TA Q2000, Newcastle, DE, USA) measurements were used to characterize the thermophysical properties of the CPCMs with the heating and cooling rate of 10 K min^−1^ in a nitrogen atmosphere. The heat transfer efficiency of the samples was characterized by infrared thermal imaging spectrometer (FLUKE TiX580, Everett, WA, USA). The thermal conductivities of the composites were measured using a thermal constant analyzer (LFA 467, Selb, Germany, 1 mm in thickness and 12.7 mm in diameter of the samples).

## 3. Results and Discussion

### 3.1. Characterizations of Macroporous rGO Aerogels

**Figure 1:**
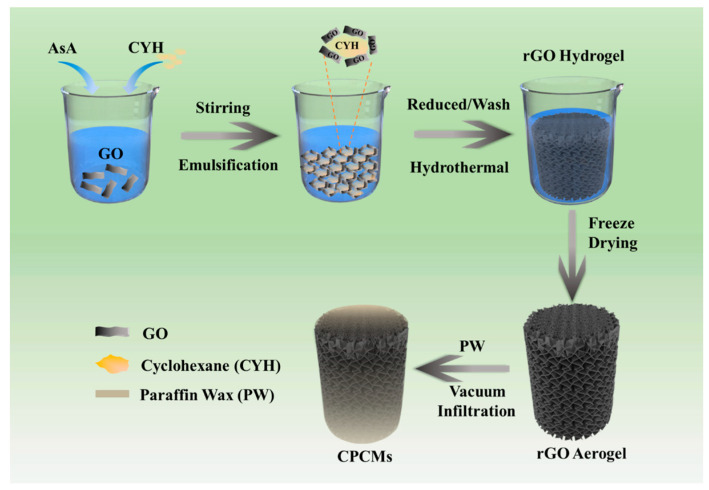
A schematic description of the preparation of the three-dimensional rGO-aerogel-based phase-change materials.

The detailed preparation process of three-dimensional macroporous rGO aerogels and their composite phase-change materials (CPCMs) are schematically described in Figure 1. The prefabricated GO nanosheets are steadily dispersed at the interface of water and cyclohexane (CYH) through emulsification by high-speed ultrasonic agitation. The GO nanosheets wrapped in CYH droplets are bonded to form rGO hydrogels by reduction of ascorbic acid (AsA) and hydrothermal reaction. After repeated rinsing and freeze-drying, the three-dimensional macroporous rGO aerogel is obtained. And finally, the phase change unit of PW is loaded into the pores of rGO aerogel to obtain rGO/PW composite PCMs via vacuum infiltration.

**Figure 2:**
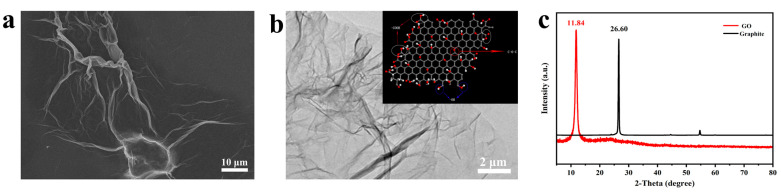
(**a**,**b**) SEM and TEM images of GO; (**c**) XRD patterns of GO and graphite.

The morphologies and structures of the prefabricated GO nanosheets and macroporous rGO aerogels are characterized by SEM and TEM. As shown in Figure 2a,b, the prefabricated GO presents a lamellar morphology and appears with a typical wrinkled texture; the lengths of GO nanosheets are about tens of microns in size observed from the TEM image. The dispersion of GO nanosheets is mainly due to the rich oxygen-containing functional groups on the graphene surface, which reduces the van der Waals forces between the layers. As shown in the inset of Figure 2b, the oxygen-containing functional groups anchored on the graphene surface are conducive to the uniform distribution of subsequent GO nanosheets in the solvent. X-ray diffraction (XRD) patterns, as shown in Figure 2c, demonstrate the acquisition of GO by the peeling of graphite. The strong peak at 2θ of 26.6° corresponds to the (002) lattice plane of graphite [24]. The diffraction peak at 11.84° of the sample is assigned to the (001) lattice plane of GO, which represents the successful preparation of GO sheets [25]. Meanwhile, the FTIR analyses also presented verify the chemical structure of the prefabricated GO; as shown in Figure S1, the peaks appearing at 1730 cm^−1^ and 1620 cm^−1^ correspond to the C=O and C=C stretching vibration peaks, and the peak at 1050 cm^−1^ is ascribed to the C–O stretching vibration [26,27]. These characteristic peaks are due to the oxygen-containing functional groups on the surface of graphene, which indicate that the graphite has been oxidized and the GO sheets are successfully obtained.

**Figure 3:**
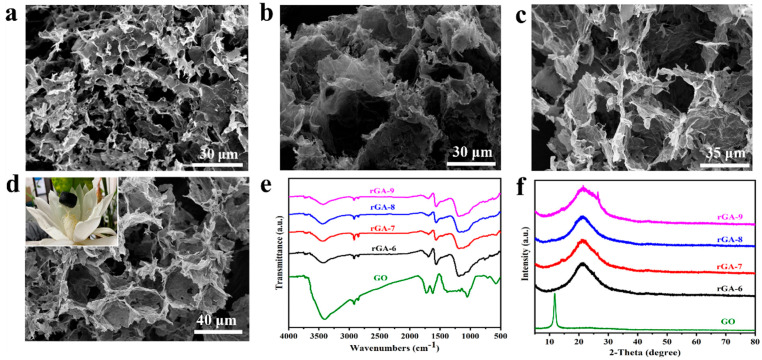
SEM images of macroporous rGO aerogels (image (**a**–**d**) represent rGA-6, rGA-7, rGA-8, and rGA-9, respectively); FT-IR spectra of rGO aerogels and GO (**e**); XRD patterns of rGO aerogel samples (**f**). (The inset of d is a digital photograph of rGA-9 aerogel resting stably above the flower buds of the epiphyllum).

With GO as the interface material, the microscopic morphologies of three-dimensional rGO porous aerogel materials obtained by the emulsification method are shown in Figure 3a–d. In order to further analyze the influence of the GO content in the emulsion on the structure of the three-dimensional porous aerogel, different amounts of GO are added to the reaction system, and depending on the GO content, the corresponding obtained rGO aerogels shown in Figure 3a–d are named rGA-6, rGA-7, rGA-8, and rGA-9, respectively. All the rGO aerogel samples reveal three-dimensional porous network morphologies, and the pores mostly present macroporous structures, which are beneficial to encapsulate the PCM and maintain a high phase-change enthalpy. Additionally, by comparing the SEM images of rGA-6, rGA-7, rGA-8, and rGA-9, the pore structure shows certain regular changes according to the added amount of GO. The sample of rGA-6, as shown in Figure 3a, exhibits a non-continuous three-dimensional network structure, and the morphology of droplet holes is not obvious. For rGA-7 and rGA-8 displayed in Figure 3b,c, the samples already have prototypes of three-dimensional network structures, and the pores are cross-linked and tightly integrated. However, the pore structure of the samples also reveals disorder and the pore size is not uniform. As the GO content increased, the rGA-9 sample showed a relatively regular and dense three-dimensional macroporous network structure, and the average pore size was about 10~20 μm. Simultaneously, the digital photo of the rGA-9 aerogel, as shown in the inset of Figure 3d, reveals that the sample can rest stably above the flower buds of the epiphyllum, which means that the rGA-9 network has a small density and a high porosity and the detailed density value is about 0.09 g/cm^3^ calculated by weighing the mass and volume of the sample. The macroporous 3D rGA aerogels with appropriate pore size distribution and pore structure will provide high porosity, pore volume, and sufficient capillary force, which is conducive to encapsulating PCMs and improving the energy storage capacity of composite PCMs. The effect of pore structure on thermal storage performance will be studied in detail in a later section.

The FT-IR spectrum of rGO aerogels and GO are performed to analyze the chemical structure. The spectra of rGA-6, rGA-7, rGA-8, and rGA-9, as shown in Figure 3e, the rGO aerogels have similar characteristic peaks and without any obvious peak change with the difference in GO content. This suggests that the GO content does not affect the chemical composition of the rGO aerogel. The peaks in the rGO aerogels appearing at 1730 cm^−1^ and 1620 cm^−1^ that are the same as the GO sample represent the C=O and C=C stretching vibration peaks, respectively [28,29]. However, by comparing with the GO sample, the peak intensity of the rGO aerogels has been significantly reduced and, meanwhile, the peak at 1050 cm^−1^ ascribed to the C–O stretching vibration disappeared in the rGO aerogels, indicating that the oxygen-containing functional groups are removed, and the GO is successfully reduced [24]. XRD patterns are also employed to investigate the structural evolution of the aerogels (Figure 3f). The samples of rGA-6, rGA-7, rGA-8, and rGA-9 show a similar broad peak at 2θ of 22.36°, which is assigned to the (002) lattice planes of rGO; at the same time, the strong peak at 11.84°, which belongs to the (001) lattice plane of GO vanished in the rGO aerogels [30]. This demonstrates the successful reduction in GO material during the process of constructing three-dimensional macropores rGO aerogels.

**Figure 4:**
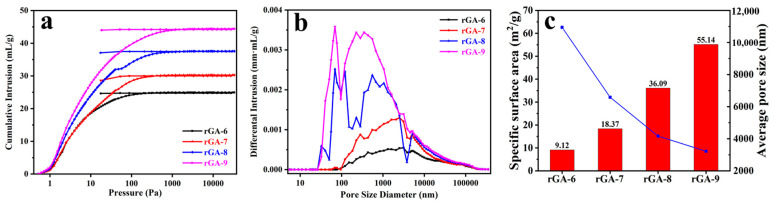
(**a**) Mercury intrusion and extrusion curves of rGO aerogels. (**b**) Pore size distribution curves. (**c**) Macropores structure parameter diagram of rGO aerogels by mercury intrusion method.

The pore structure and pore size as the critical parameters for the aerogel are further confirmed by the mercury intrusion method; as shown in Figure 4a, the mercury intrusion and extrusion curves of rGA-6, rGA-7, rGA-8, and rGA-9 present a certain pattern that the volume of cumulative intrusion is rising with the increased amount of GO for the reaction system. In addition, it is interesting that the mercury extrusion curves do not close with intrusion curves, indicating that the pores structure of the aerogel can limit the flow of intruded liquid mercury, which is conducive to preventing leakage after the PCM are loaded into the pores of rGO aerogels. This characteristic has a profound influence on the CPCMs with high energy storage capacity. The pore size distribution curves (Figure 4b) show that the pore size of the samples mainly ranges from 100 nm to 10 μm. With the increase in GO content, the pore size distribution of samples becomes more concentrated, and the corresponding average pore size also decreases, as listed in Table 1. This is because the increase in GO content makes it evenly and sufficiently distributed at the interface of the emulsion and leads to a more stable emulsion in the system. Thus, dense and uniform pores are obtained, accompanied by the removal of emulsion droplets. Additionally, it can be found by analyzing and comparing the data in Figure 4c and Table 1 that the surface area of rGA-6, rGA-7, rGA-8, and rGA-9 shows an increasing trend from 9.12 m^2^/g to 55.14 m^2^/g and at the same time, the average pore size of the samples is decreased from 10.94 μm to 3.22 μm. This demonstrates well that the size of pores in the aerogel is adjustable according to the added amount of GO nanosheets, and the increase in GO content makes the three-dimensional network structure of aerogel more compact and uniform. Interestingly, the porosity of the four samples did not change significantly as the GO content changed, and the range of porosity was mainly at about 95%. This is because, for rGO aerogel, the porosity is mainly contributed by macroporous channels, the density of the aerogel is small, and the porosity is relatively large; subtle changes in GO have no substantial effect on the porosity of the whole aerogel. As is well known, for aerogel porous materials, proper internal pore size has a great influence on the overall properties of CPCM. Excessively small channels will restrict the entry of the PCM and result in the enthalpy value being unsatisfied in the CPCM. But as the pore size is too large, the insufficient capillary force provided by the pores will cause the leakage of CPCM, and simultaneously the thermal conductivity of the CPCM will be correspondingly reduced. Therefore, changing the amount of GO in the emulsion system to adjust the pore structure and pore size of rGO aerogel is a meaningful method to improve the comprehensive properties of CPCMs.

### 3.2. Leakage-Proof and Thermophysical Properties of rGA/PW Composites

**Figure 5:**
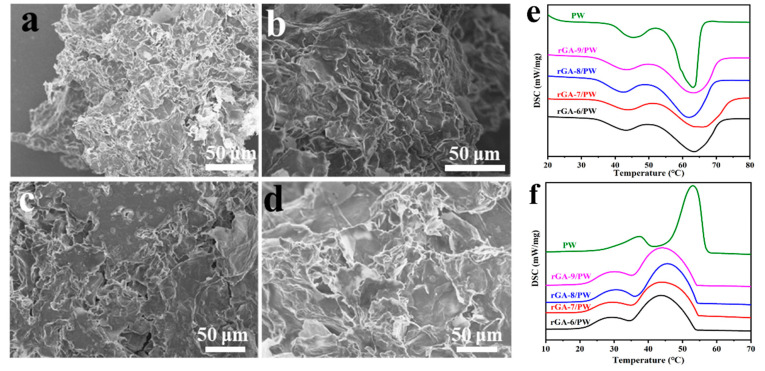
(**a**–**d**) SEM images of rGA-6/PW, rGA-7/PW, rGA-8/PW, and rGA-9/PW, respectively; (**e**,**f**) DSC curves of samples.

The rGO-based composite PCMs (rGA/PW) are prepared by encapsulating PW in rGO aerogels using a vacuum infiltration strategy. For rGA-6, rGA-7, rGA-8, and rGA-9 macropores supports, the PW is successfully adsorbed and stabilized in the three-dimensional porous network of rGO aerogels, and the pores of the aerogel become smaller or even disappeared, as shown in Figure 5a–d. According to differences in rGO aerogels, the corresponding rGO-based CPCMs are named rGA-6/PW, rGA-7/PW, rGA-8/PW, and rGA-9/PW, respectively. The XRD patterns and FT-IR spectra of rGA/PW are presented in Figures S2 and S3. It can be seen from the XRD patterns that rGA-6/PW, rGA-7/PW, rGA-8/PW, and rGA-9/PW samples have similar diffraction peaks at 21.4°, 23.7°, 35.8°, 40.2°, respectively, and 42.2° as PW, and without any new diffraction peak generated [31]. This is due to strong diffraction peaks in PW, which leads to the peak in rGO aerogel being covered at 21.5°. It also indicates that the recombination of rGO aerogel and PW is a physical process that does not affect the chemical characteristics of each component. For the FT-IR spectra, similarly, the CPCMs have the characteristic peaks of both rGO and PW, and no new peaks are formed. This further demonstrates that the formation of CPCMs is physical adsorption.

**Figure 6:**
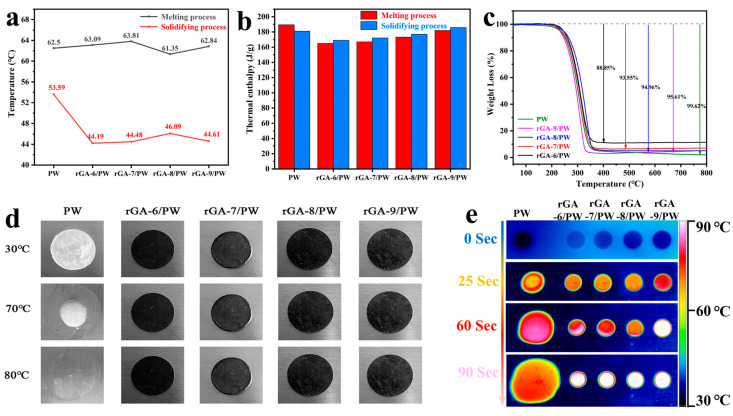
(**a**) Peak temperature at phase-transition period of rGA/PW and PW. (**b**) Enthalpy histogram of melting and solidifying process. (**c**) TGA curves of samples. (**d**) The digital photos of leak test. (**e**) Infrared thermal images of PW, rGA-6/PW, rGA-7/PW, rGA-8/PW, and rGA-9/PW that are heated on a hot stage of 90 °C.

T Thermophysical properties of rGA/PW are investigated by the DSC curves, including the melting and freezing processes. As shown in Figure 5e,f, rGA-6/PW, rGA-7/PW, rGA-8/PW, and rGA-9/PW CPCMs have similar melting DSC curves to PW with two phase transition peaks. The left weak peak at low temperature corresponds to the solid–solid phase transition of PW, while the strong peak with larger latent heat corresponds to the solid–liquid transition of PW [32]. However, for the solidifying process, the DSC curves of rGA/PW samples present some difference with pristine PW in the phase transition temperature. The peak phase-change temperature during the solidifying process has dropped by an average of 5 °C, and the specific data of peak phase-change temperature during the melting and solidifying processes are shown in Figure 6a. This is due to the abundant intermolecular force between PW and rGO aerogel, which increases the crystallized nuclear energy of liquid paraffin and finally leads to the crystallization and solidification processes happening at lower temperatures [33]. This feature can be utilized to regulate the solidification temperature of CPCMs by adjusting the interaction between rGO and PW. Latent heat, as a critical property of CPCMs, plays an important role in obtaining high-performance PCMs. As shown in Figure 6b, the latent heat histogram of the melting and solidifying processes exhibits that the rGA/PW have close endothermic and exothermic values to those of pristine PW. And with the increase in rGO content in the aerogel, the latent heat value of the CPCMs also increases gradually. This is attributed to the higher uniformity of the three-dimensional network structure and more concentrated pore size of rGO aerogel, which increases the capillary force and prevents leakage of melting PW. Thus, the thermal storage capacity of the CPCMs is improved, accompanied by the increase in rGO content in the aerogel supports. This also proves that the thermal storage performance of CPCM can be effectively improved by regulating the three-dimensional pore structure and pore size distribution of rGO aerogels.

Thermal stability and component composition of the CPCMs are analyzed by TGA curves; as shown in Figure 6c, the PW shows almost complete thermal decomposition from 200 °C to 800 °C under a nitrogen atmosphere. The mass loss of CPCMs samples of rGA/PW varied from 250 °C to 800 °C depending on the rGO aerogel supports. The rGA-6/PW, rGA-7/PW, rGA-8/PW, and rGA-9/PW CPCMs present excellent thermal stability below 200 °C, which is important for practical application. With the temperature rising from 200 °C, all the samples show an obvious mass reduction, and after the temperature reaches above 450 °C, the quality of the samples remains stable gradually. The final mass losses of rGA-6/PW, rGA-7/PW, rGA-8/PW, and rGA-9/PW are 88.85%, 93.55%, 94.96% and 95.61%, respectively. Considering the thermal stability of rGO under a nitrogen atmosphere, the mass loss of the CPCMs is mainly due to the thermal decomposition of PW, which is consistent with the calculation result of PCM loading according to Equation (1), that is, the mass fraction of PW in CPCMs is 88%, 93%, 95%, and 96%, corresponding to rGA-6, rGA-7, rGA-8, and rGA-9 three-dimensional rGO aerogel supports, respectively. The main reason for the difference in PCM loading is the adequacy of porosity and interaction forces between PW and rGO aerogel supports. For the rGA-6/PW, rGA-7/PW, and rGA-8/PW samples, their pore structure is not regular and tightly connected compared to rGA-9/PW and the pore size is too large to provide sufficient capillary force to restrict the flow of liquid PCM, resulting in leakage of PW and differences in the final amount of PCM loading. These results are also consistent with the analysis of the morphology and pore structure of the rGA supports.

The detailed thermophysical data of melting and solidifying enthalpy (ΔH_m_ and ΔH_s_), peak temperature at phase change period (T_m_ and T_s_), loading mass fraction of PW in CPCMs, and the CPCMs melting enthalpy ratio to PW are listed in Table 2. It can be found that the loading fraction of PW in the CPCMs is consistent with the melting enthalpy ratio to PW. Meanwhile, for the rGA-6/PW, rGA-7/PW, rGA-8/PW, and rGA-9/PW samples, the loading fraction of PW is gradually increased with the rise in rGO content in the aerogel supports, and the latent heat value of the CPCMs also increases. These trends are consistent with the DSC analysis and the characterization results of pore structure.

The shape-stability and leakage-proof properties of rGA/PW are verified by placing the sample on a heating platform at 90 °C; as illustrated in Figure 6d,e, the status of samples is recorded with digital photos and infrared imager. When the samples of rGA-6/PW, rGA-7/PW, rGA-8/PW, rGA-9/PW (black disc), and PW (translucent disc) are just placed on the heating platform, the corresponding samples’ surface temperatures are all about 30 °C, which are closed to the ambient temperature (Figure 6d) and the shape of the samples kept steady. With the extension of the time, the surface temperature of the samples rises to about 70 °C, and the shape of PW starts to melt and accompany the volume change. But for GA-6/PW, rGA-7/PW, rGA-8/PW, and rGA-9/PW samples, the shape has remained constant. Finally, as the temperature increases up to about 80 °C, the PW melts completely and becomes transparent, and the other samples remain stable in shape. Additionally, the temperature changes in the upper surface of each sample are synchronously reflected in the infrared image (Figure 6e). The same color of samples and background are observed when the samples are first placed on the heating platform. After 25 s of heating, the colors of all the samples changed; the color of PW changed to blond, and the GA-6/PW, rGA-7/PW, and rGA-8/PW changed to yellow. The most obvious color change happened in rGA-9/PW, which changed to red. This indicates that the upper surface temperature of rGA-9/PW changes fastest. The main reason for this phenomenon is that the rGA-9 three-dimensional macroporous network supports with regular and dense pore structure form continuous fast heat transfer channels inside the composite PCMs of rGA-9/PW, accelerating the rate of heat conduction from the bottom to the top, which also indicates that this sample has higher thermal conductivity. With the extension in heating time, the color of all the CPCMs changes to bright white, but the color of the PW sample is still orange, and the shape deformation is obvious. The infrared image not only indicates the excellent shape-stabilized performance of rGA/PW CPCMs but also demonstrates the higher thermal transfer efficiency of rGA-9/PW than other samples. Consequently, on the one hand, the three-dimensional porous rGO aerogel supports obtained by emulsification of water and oil can encapsulate PW stably and efficiently; on the other hand, via controlling the content of rGO to adjust the pore structure of aerogel supports can achieve simultaneous improvement in heat storage capacity and heat transfer performance of CPCMs.

### 3.3. Thermal Conductivity and Cyclic Stability Characterization of CPCMs

**Figure 7:**
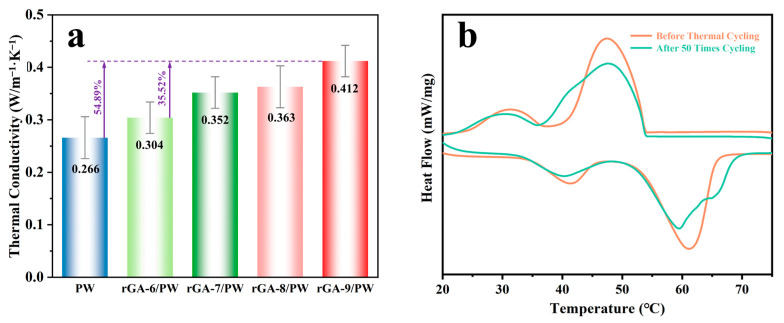
(**a**) Bar diagram of thermal conductivity of samples. (**b**) DSC curves of rGA-9/PW before and after 50 times thermal cycling.

In addition to the high thermal storage capacity and shape-stabilized properties, thermal conductivity is also a crucial property for CPCMs. As can be seen from the aforementioned infrared images, the heat transfer efficiency of CPCMs is higher than that of PW. In order to quantify the heat transfer properties, the thermal conductivity is measured for rGA/PW CPCMs and PW, as shown in Figure 7a, pristine PW with the thermal conductivity of 0.266 W/m^−1^·K^−1^. In particular, the thermal conductivities of CPCMs are 0.304 W/m^−1^·K^−1^, 0.352 W/m^−1^·K^−1^, 0.363 W/m^−1^·K^−1^ and 0.412 W/m^−1^·K^−1^ for the GA-6/PW, rGA-7/PW, rGA-8/PW, and rGA-9/PW samples, respectively, which produce a significant improvement than PW. Compared with PW, the thermal conductivity increase in the rGA-9/PW sample reaches 54.89%, and with the increase in graphene content in the aerogel supports, the thermal conductivity of the CPCM also presents a trend of gradual improvement. This is mainly because the rGO nanosheets have excellent in-plane thermal conductivity; thus, the increase in rGO content in CPCMs can improve the overall heat transfer efficiency of the system. In addition, the pore structure of rGO aerogel supports is more continuous and uniform, which is conducive to the migration of phonons in the process of heat conduction [34]. Meanwhile, the higher loading fraction of PW and relatively uniform network structure can increase the adhesion energy between the supporting materials and PW, which is beneficial to reducing the phonon scattering and improving the heat conduction performance [35].

The reliability and stability of CPCMs after melting and solidifying thermal cycling also are extremely important for sustainable application. The DSC curves of rGA-9/PW CPCMs before and after 50 melting and solidifying cycles are shown in Figure 7b. It can be found that no obvious changes happened, including the peak shape and peak position before and after 50 melting and solidifying cycles. And the melting enthalpy values of CPCMs are collected in Table 3 before and after circulation. The melting enthalpy remaining still can reach above 90% after 50 times thermal cycling, indicating that the rGA/PW CPCMs have excellent thermal stability. This extraordinarily stable phase transition performance is assigned to the sufficient capillary force and three-dimensional encapsulation characteristics provided by crosslinked rGO aerogel supports.

To sum up, the three-dimensional macroporous rGO aerogel provides sufficient porosity and capillary force to obtain high thermal storage capacity and shape-stabilized CPCMs. Meanwhile, the continuous crosslinked network structure of rGO aerogel supports endows highly efficient thermal transport pathways for the CPCMs. In addition, the pore size distribution and pore structure of rGO aerogel supports can be adjusted by regulating the GO content, which is conducive to the simultaneous improvement in the thermal storage density and thermal transfer efficiency of CPCMs. Therefore, reasonable utilization of the rGO aerogel supports can effectively enhance the comprehensive properties and expand its potential applications of rGA/PW CPCMs.

## 4. Conclusions

In summary, three-dimensional macroporous rGA supports with adjustable pore structures are prepared by the water–oil emulsion template method and hydrothermal reduction process. The shape-stabilized composite PCMs of rGA/PW are constructed after the combination of 3D macroporous rGA supports and paraffin wax (PW) through vacuum melting infiltration. After regulating the pore structure of rGA supports by changing the GO content, the obtained rGA-9/PW composite PCMs achieve excellent thermal storage and thermal transfer performance with a phase-change enthalpy of 179.94 J/g and an enhanced thermal conduction of 0.412 W/m^−1^·K^−1^, 54.89% higher than pristine PW and enduring thermal cycling stability. The prepared 3D macroporous rGA-based composite PCMs with high thermal storage capacities and fast heat transfer properties will effectively expand the application of PCMs in the field of thermal energy storage in the future.

## Figures and Tables

**Table 1:** Pore size and surface area parameter of rGO aerogels by mercury intrusion method.

Samples	rGA-6	rGA-7	rGA-8	rGA-9
Surface Area (m^2^/g)	9.12	18.37	36.09	55.14
Average Pore size (μm)	10.94	6.59	4.16	3.22
Porosity (%)	95.71	94.51	95.35	96.58

**Table 2:** Phase-change parameters of PW and rGA/PW.

Materials	Loading Fraction	Melting Enthalpy Ratio to PW	Melting		Solidifying	
			Peak T_m_ (°C)	ΔH_m_ (J/g)	Peak T_s_ (°C)	ΔH_s_ (J/g)
Pure PW	100%	100%	100%	55.4	185.3	50.1
rGA-6/PW	88.85 wt%	88.99%	88.99%	63.09	164.90	44.19
rGA-7/PW	93.55 wt%	90.09%	90.09%	63.81	166.95	44.48
rGA-8/PW	94.96 wt%	93.50%	93.50%	61.35	173.26	46.09
rGA-9/PW	95.61 wt%	97.10%	97.10%	62.84	179.94	44.61

**Table 3:** Melting enthalpy change parameters of samples before and after circulation.

Samples	Enthalpy before Circulation (J/g)	Enthalpy after Circulation (J/g)	Enthalpy Remaining (%)
rGA-6/PW	164.90	152.43	92.43
rGA-7/PW	166.95	153.16	91.74
rGA-8/PW	173.26	155.64	89.83
rGA-9/PW	179.94	165.14	89.00

## Data Availability

Restrictions apply to the availability of these data. Data are available from the authors.

## Supplementary Materials

The following supporting information can be downloaded at: https://www.mdpi.com/article/10.3390/ma16134878/s1, Figure S1. FT-IR spectra of GO; Figure S2. XRD patterns of (a) rGA-6, PW, rGA-6/PW; (b) rGA-7, PW, rGA-7/PW; (c) rGA-8, PW, rGA-8/PW; (d) rGA-9, PW, rGA-9/PW; Figure S3. FT-IR spectrum of (a) rGA-6, PW, rGA-6/PW; (b) rGA-7, PW, rGA-7/PW; (c) rGA-8, PW, rGA-8/PW; (d) rGA-9, PW, rGA-9/PW.
