# Supplementary material for: Three-Dimensional Macroporous rGO-Aerogel-Based Composite Phase-Change Materials with High Thermal Storage Capacity and Enhanced Thermal Conductivity
Source: Materials (Basel). 2023 Jul 7;16(13):4878. doi: 10.3390/ma16134878 (PMC10343296; doi:10.3390/ma16134878)
Supplement: Supplementary file 1 [file materials-16-04878-s001.zip › materials-2464022-supplementary.docx]

Three-Dimensional Macroporous rGO-Aerogel-Based Composite Phase-Change Materials with High Thermal Storage Capacity and Enhanced Thermal Conductivity

**Zhang Tao ^1^, Wei He ^2^, Xiaoliang Xu ^3^, Jianzhong Fan ^1^, Zhifeng Zhang ^1^,
Ziyue Yang 1, Yanqiang Liu ^1,^*, Heng Ma ^4^, Miao Qian ^4^ and Mu Yang ^3,^***

^1^ GRINM Metal Composites Technology Co., Ltd., Beijing 101407, China

^2^ Wuhan NARI Limited Liability Company, State Grid Electric Power Research Institute, Wuhan 430074, China

^3^ Beijing Advanced Innovation Center for Materials Genome Engineering, Beijing Key Laboratory of Function Materials for Molecule & Structure Construction, School of Materials Science and Engineering, University of Science and Technology Beijing, Beijing 100083, China

^4^ Zhejiang Huadian Equipment Testing Institute Co., Ltd., Hangzhou 310015, China

* Correspondence: liuyanqiang@grinm.com (Y.L.); yangmu@ustb.edu.cn (M.Y.)


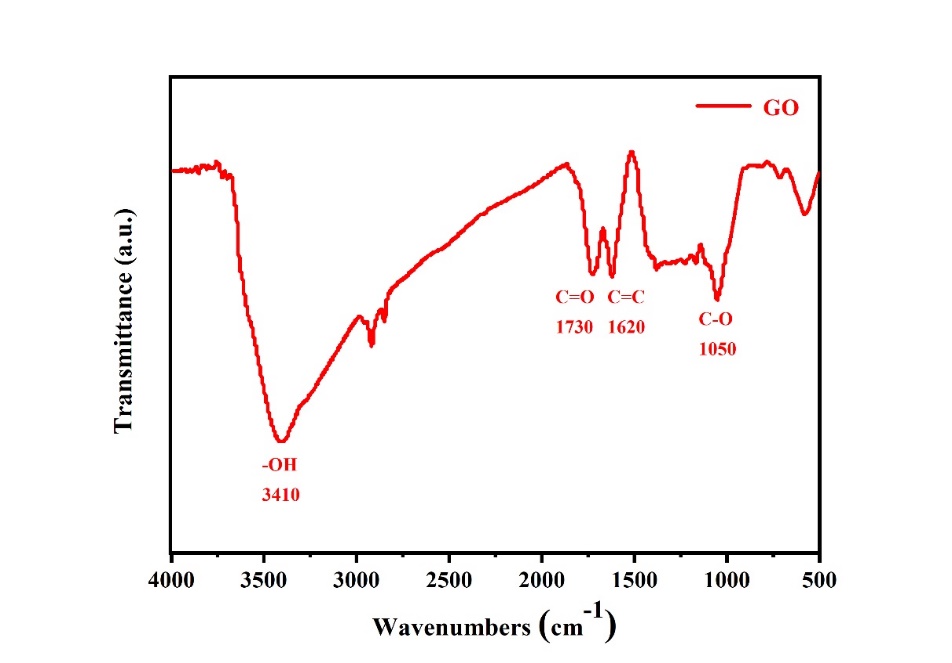


**Figure S1. FT-IR spectra of GO.**


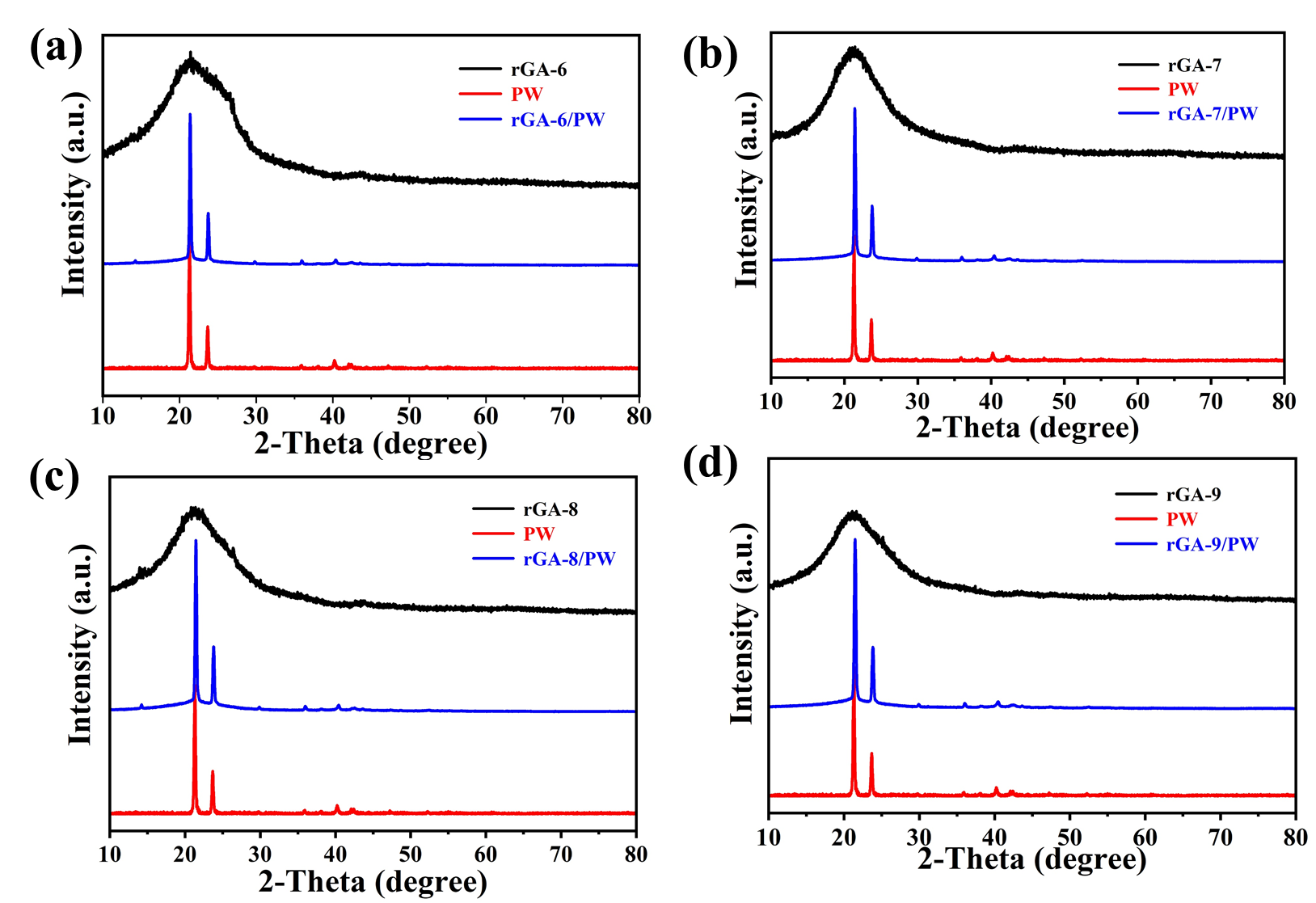


**Figure S2. XRD patterns of (a) rGA-6, PW, rGA-6/PW; (b) rGA-7, PW, rGA-7/PW; (c) rGA-8, PW, rGA-8/PW; (d) rGA-9, PW, rGA-9/PW.**


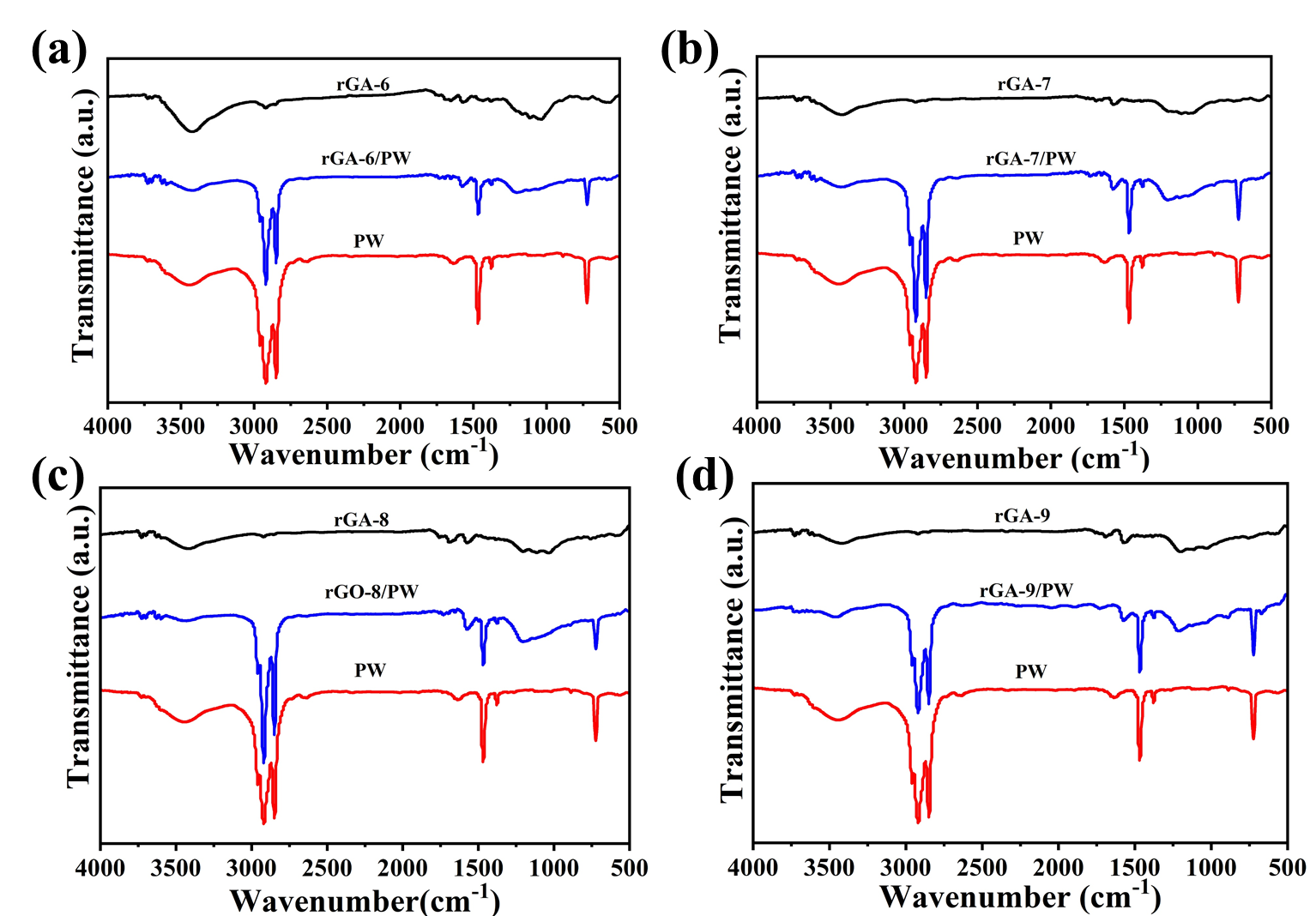


**Figure S3. FT-IR spectrum of (a) rGA-6, PW, rGA-6/PW; (b) rGA-7, PW, rGA-7/PW; (c) rGA-8, PW, rGA-8/PW; (d) rGA-9, PW, rGA-9/PW.**
